# Deep Sleep and Parietal Cortex Gene Expression Changes Are Related to Cognitive Deficits with Age

**DOI:** 10.1371/journal.pone.0018387

**Published:** 2011-04-04

**Authors:** Heather M. Buechel, Jelena Popovic, James L. Searcy, Nada M. Porter, Olivier Thibault, Eric M. Blalock

**Affiliations:** Department of Molecular and Biomedical Pharmacology, University of Kentucky College of Medicine, Lexington, Kentucky, United States of America; Nathan Kline Institute and New York University School of Medicine, United States of America

## Abstract

**Background:**

Age-related cognitive deficits negatively affect quality of life and can presage serious neurodegenerative disorders. Despite sleep disruption's well-recognized negative influence on cognition, and its prevalence with age, surprisingly few studies have tested sleep's relationship to cognitive aging.

**Methodology:**

We measured sleep stages in young adult and aged F344 rats during inactive (enhanced sleep) and active (enhanced wake) periods. Animals were behaviorally characterized on the Morris water maze and gene expression profiles of their parietal cortices were taken.

**Principal Findings:**

Water maze performance was impaired, and inactive period deep sleep was decreased with age. However, increased deep sleep during the active period was most strongly correlated to maze performance. Transcriptional profiles were strongly associated with behavior and age, and were validated against prior studies. Bioinformatic analysis revealed increased translation and decreased myelin/neuronal pathways.

**Conclusions:**

The F344 rat appears to serve as a reasonable model for some common sleep architecture and cognitive changes seen with age in humans, including the cognitively disrupting influence of active period deep sleep. Microarray analysis suggests that the processes engaged by this sleep are consistent with its function. Thus, active period deep sleep appears temporally misaligned but mechanistically intact, leading to the following: first, aged brain tissue appears capable of generating the slow waves necessary for deep sleep, albeit at a weaker intensity than in young. Second, this activity, presented during the active period, seems disruptive rather than beneficial to cognition. Third, this active period deep sleep may be a cognitively pathologic attempt to recover age-related loss of inactive period deep sleep. Finally, therapeutic strategies aimed at reducing active period deep sleep (e.g., by promoting active period wakefulness and/or inactive period deep sleep) may be highly relevant to cognitive function in the aging community.

## Introduction

Age-related cognitive deficits are a highly prevalent and important health risk in the human population (reviewed in [1]), can presage development of age-related neurodegenerative disease [2], [3], [4], and are a primary reason for elderly placement in assisted living facilities [5]. Sleep dysregulation is also a common complaint among the elderly. During the night, the constellation of age-related sleep changes include circadian advance, sleep fragmentation, insomnia [6], [7], [8], [9], [10], [11], and loss of deep, slow wave sleep [9], [12], [13], while daytime symptoms include sleepiness, increased napping and breakthrough sleep. Further, healthy younger adults exposed to experimentally induced selective deprivation of night time (inactive period) deep sleep show some aging-like phenotypes, including daytime sleepiness [14], blood chemistry changes similar to those seen in metabolic syndrome (a potential precursor to the development of type II diabetes) and cognitive deficits [15], [16], [17].

Although clearly vital to normal function, sleep is only grudgingly yielding to scientific inquiry regarding its role(s) in physiology. Recent studies suggest that deep, slow wave sleep during the inactive period promotes memory [18], [19], [20], [21], possibly through localized synaptic [22], [23], [24] and macromolecular synthesis [25] effects. Thus, the dysregulated slow wave sleep seen with age might contribute to cognitive deficits seen with aging. Despite the seemingly similar effects of age and sleep dysregulation on cognition, and the high prevalence of sleep changes with age, relatively few studies have investigated possible mechanistic links between sleep architecture changes and age-related cognitive decline.

Here, we used the F344 rat model of aging to investigate this relationship. Young and aged rats were surgically implanted with wireless telemetry devices in order to measure sleep architecture. Each subject was evaluated for cognitive performance on the Morris water maze. Further, microarray analysis assessed potential molecular relationships among aging, behavior, and sleep in brain tissue. Because sleep stages have been reported to be brain region specific, we selected parietal cortex for array analysis as it was closest to the recording electrodes (and therefore hypothetically most germane to correlations with sleep measures).

## Materials and Methods

### Subjects

Young adult (3 mo) and aged (21 mo) male Fischer 344 rats obtained from the NIA aging colony were individually housed with crinkled paper bedding and a cardboard tube. Animals were maintained on a 12:12 light/dark cycle in the housing facility and were given access to food and water ad-libitum. All animals were evaluated for pathology (e.g., pituitary and mammary tumors, splenomegaly, and cataracts). Two aged subjects were excluded (one with a pituitary tumor, one with a mammary tumor) leaving n = 9 young and n = 9 aged for subsequent analyses.

#### Ethics statement

This study was carried out in strict accordance with the recommendations in the Guide for the Care and Use of Laboratory Animals of the National Institutes of Health. The protocol was approved by the University of Kentucky Office of Research Integrity Institutional Animal Care and Use Committee. Surgical (isoflurane) and euthanatising (CO_2_) anesthesia were used and all efforts were made to minimize suffering.

### Surgery

All subjects were implanted with wireless EEG/EMG emitters according to standard procedures (Data Sciences International- TL11M2-F40-EET). Briefly, animals were anesthetized with isoflurane and placed in a stereotaxic frame. A two inch incision was made to expose the skull and spinotrapezius muscles. The emitter was placed under the skin between the left scapulae and the left ileum along the flank. The exposed dorsal region of skull was cleaned with 3% peroxide and the skull surface dried with sterile cotton swabs soaked in 70% ethanol. A 0.7 mm hole was drilled 1 mm from either side of the sagittal suture line and 1–2 mm anterior to the lambda suture line for the EEG leads. EEG leads were bent into a ‘u’ shape and the base of the ‘u’ inserted into the hole so that wire contacted the dura over the parietal cortex. They were then covered with dental cement and left to dry. EMG activity was recorded by surgically inserting two wire electrodes perpendicular to trapezius muscle fiber. The free wire end was capped with insulation and both sides of the incision were tied off with surgical thread to prevent fluid infiltration into the insulation. The incision was then closed with 4–6 mattress stitches. Immediately after surgery, data was collected from the emitters implanted in the rats. This allowed us to monitor their surgery recovery and to evaluate when they had stable sleep/wake patterns. One young animals' emitter failed and his sleep architecture data is not included in the study.

### Sleep Data Acquisition and Analysis

Animals were housed individually and cages were at least 18” apart to avoid interference during radiotelemetry data acquisition. For these nocturnal rodents, the first four hours of active (dark) and inactive (light) periods on the day prior to the water maze probe trial were analyzed. Two independent researchers scored each sleep segment using Neuroscore's analysis console. EEG, EMG, temperature and locomotor activity data were recorded continuously with DSI's Data Art acquisition software and binned in 10 second epochs. Epochs were scored in 30 second increments while being viewed in both 2 minute and 5 minute windows. EEG waves were stratified into ‘low amplitude’ (≤50% of maximum) and ‘high amplitude’ (> 50% of maximum) tiers, and underwent fast Fourier transforms for each of 5 frequency ranges: Δ (0.5–4 Hz), Θ (4–8 Hz), Α (8–12 hz), Σ (12–16 Hz) and B (16–24 Hz). EMG waves were stratified into 3 tiers: ‘basal’ ≤33% (seen during REM); ‘intermediate’ (between 33% and 66%); ‘high’ (> 66%). Stages were established as follows: Wake- intermediate or high EMG ± locomotor activity, EEG variable; Light sleep- low amplitude EEG, intermediate EMG, and no locomotion; REM (paradoxical) sleep- high frequency EEG, ‘basal’ EMG and no locomotor activity; deep sleep- high amplitude EEG activity enriched in delta band frequency, basal to light EMG activity, no locomotor activity. Prior assigned sleep stages informed subsequent assignments. Ambiguous epochs, those disagreed upon by independent scorers, as well as those containing artifacts, were not scored and accounted for <5% of scored time.

### Water Maze Testing

The water maze (black circular pool, 190 cm in diameter) was placed equidistant (∼60 cm) to a continuous wall of black curtains hanging from the ceiling, making the environment relatively neutral. Three high contrast black and white cues (90 cm×90 cm, representing a circle, triangle and vertical lines), were placed on the curtains. Pool temperature was maintained at 26±2°C. One quadrant contained a 15 cm diameter escape platform covered with black neoprene for improved traction. Illumination in the room was set such that the Videomex-V water maze monitoring system (Columbus Instrument, Columbus, OH) could reliably monitor animal movements with no artifacts. The Morris water maze [26] training and probe sessions took place between 10AM and 2PM as previously published [27], [28], [29], [30]. This ‘standard operating procedure’ for water maze testing in rats occurs during their inactive period. Thus, the duration of this procedure was kept consistent (4 hours) across all subjects on each day of behavioral acquisition to control for potentially sleep disruptive influences. Prior to surgery, animals were evaluated on a visual cue task (3×60s per day, 4 days). All animals were able to swim directly to the visual platform by the 12^th^ trial (data not shown). Animals were then implanted (see above) and allowed to recover for 2 weeks. Sleep architecture and behavior were then evaluated during the third week. For maze performance, a 5 day protocol was used (day 1: visual cue trial; days 2–4: 3 trials per day, hidden platform; day 5- probe trial with platform removed). On the cue and training days, each animal began in a different quadrant on each of three trials. They were given one minute to find the platform, one minute on the platform and a two minute inter-trial interval. On the probe day, the platform was removed and each rat was given one 60 s trial. Path length and latency were measured to either the platform (trial) or a computer-superimposed silhouette of the platform (probe) (Videomex-V water maze monitoring system, Columbus Instrument).

### Tissue Collection and Microarray Analysis

Immediately after the probe trial, rats were killed by CO_2_ anesthesia and rapid decapitation. For a subset of animals selected by order of entry (the first 6 animals per age group) brains were removed and parietal cortex dissected out in chilled (0°C) artificial cerebrospinal fluid. Parietal cortex was selected for the present study to facilitate transcriptional profile correlation with slow wave activity measured during deep sleep as this was the cortical region beneath which the EEG recording electrodes were situated. Tissue was flash frozen and stored at −80°C for subsequent microarray analysis. RNeasy mini kit (Qiagen) was used to extract RNA and quality of starting RNA was measured with Agilent Bioanalyzer technology (RNA Integrity Number  = 9.4±0.1). Each sample (one per subject) underwent RNA extraction, purification, and cDNA labeling separately, as described previously [27], [28], [29], [30], [31], [32] and according to standard Affymetrix procedures. Labeled cDNA from each subject was individually hybridized to an Affymetrix rat microarray (RAE230 2.0, 31099 probe sets). All arrays passed standard Affymetrix quality control (gene expression console v. 1.1): GAPDH 3′–5′ ratio 1.05±0.03, RawQ 2.51±0.16, Background noise 70.5±4.1. Scaling factor, based on target intensity of 500, Young: 1.34±0.25, Aged: 1.27±0.20; as well as % Present- Young: 67.5±1.0, Aged: 66.6±1.9 were not significantly different across treatment groups (t-test, p>0.3). Visual inspection of residual sign images of .cel files using Affy PLM [33] revealed no major geographic defects in microarray signal intensity.

The gcRMA probe level algorithm (‘justgcRMA’ command run in Bioconductor in the R operating environment) calculated signal intensities [34], [35]. Only unique probe sets/genes with ‘A’ grade annotations and at least 3 chips with signal intensities >4.3 were retained for further analysis. Values were transferred to Excel (2007, Microsoft), Bioconductor [34], MultiExperiment Viewer [MEV, [36]] and the DAVID suite of bioinformatic tools [37] for subsequent analysis. Specific statistical procedures are outlined in Results. The signal intensity (gcRMA) and images (.cel files) have been deposited to the MIAME compliant Gene Expression Omnibus (GEO) database [[38] - accession #GSE24515].

## Results

### Water Maze

**Figure 1:**
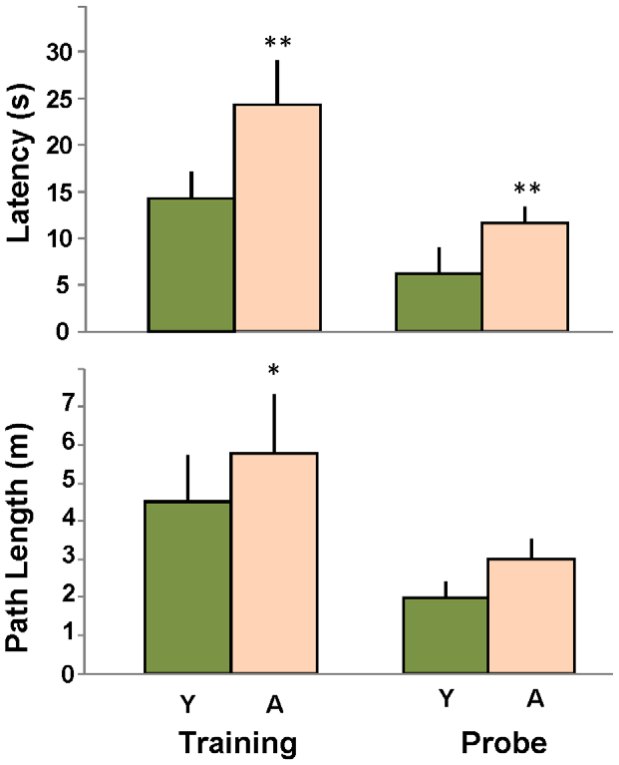
Aged rats show a deficit in performance on the Morris water maze. **Upper:** Time to reach the platform (escape latency) averaged over training (left), or time to reach platform annulus during probe trial (right). **Lower:** Path length to reach platform during training (left) or platform annulus during probe trial (right). (* p≤0.05; ** p≤0.01; 2-ANOVA repeated measures, post-hoc pairwise Fisher's protected Least Significant Difference).

Animals were trained in the Morris water maze task 3 times per day for 3 days, and administered a probe trial on the 4^th^ day (see Methods). Escape latencies across all 9 training trials were averaged for each animal and then treated as a single observation for that subject. As shown in Figure 1 (upper left) aged rats took significantly longer to find the hidden platform during training. Aged rats also took significantly longer than their younger counterparts (latency; Fig. 1; upper right) to reach the goal annulus during the probe trial. Similar age-related deficits were revealed by escape path length measurements (Fig. 1, lower panels, significant in training, trend to significant p<0.15 in probe trial). These data confirm numerous previous studies in which aged animals do not perform as well as young (e.g.,[27], [29], [39], [40]). There was no significant difference in swim velocity between young and aged subjects (probe trial: young 28.6±2.3 cm/sec; aged 28.4±3.8 cm/sec; p = 0.96; ttest), suggesting that an age-related change in swim speed did not account for increased latency.

### Sleep Architecture

**Figure 2:**
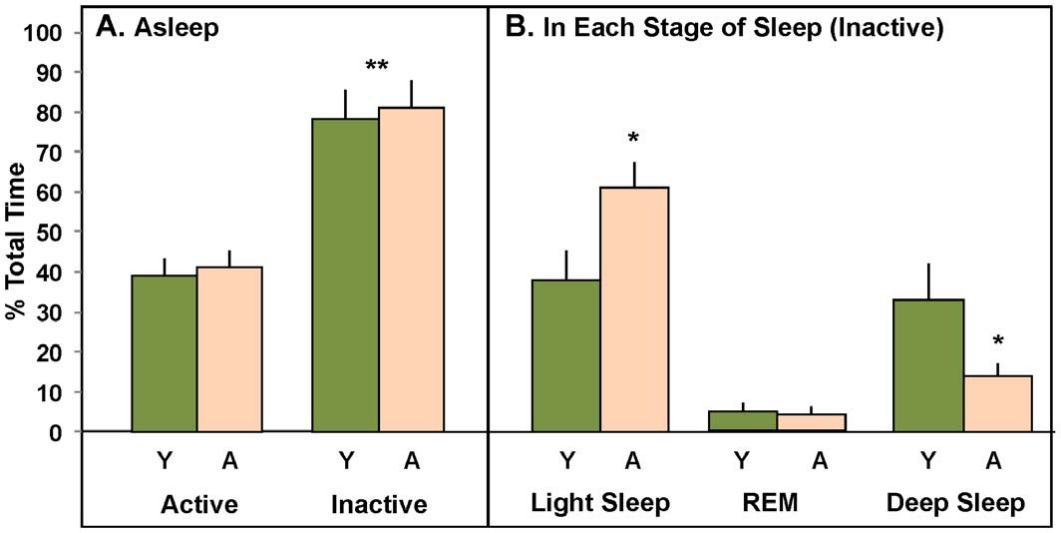
Selective loss of deep slow wave sleep with age. EEG, EMG, temperature, and locomotion radiotelemetry were recorded from young and aged subjects. **A**. % time asleep plotted as a function of age for 4 h blocks of the active vs. inactive periods. A significant increase in time asleep during the inactive period (** p<0.01, 2-ANOVA main effect of period), but no effect of age or interaction, was seen. **B**. Sleep staging analysis from first 4 hours of inactive period- staged as ‘light’ (low frequency, low amplitude EEG activity, basal – intermediate EMG activity), REM (rapid eye movement/paradoxical - high frequency, low amplitude EEG activity, basal EMG activity), and ‘deep’ sleep (low frequency, high amplitude EEG activity, basal – intermediate EMG activity- see Methods for complete description of staging analysis). Aged animals showed a significant increase in light sleep, no significant difference in REM sleep, and a significant decrease in deep slow wave sleep compared to their younger counterparts (repeated measures 2-ANOVA with a significant main effect of sleep stage [p<0.001] and interaction [p<0.001], with significant [* p<0.05] post-hoc Tukeys pairwise comparisons across age within deep sleep).

Each animal in the study was surgically implanted with a wireless EEG/EMG/temperature/locomotion telemetry system (see Methods) at least 3 weeks prior to the water maze study. To evaluate potential changes in sleep architecture (duration and intensity of different stages of sleep) with age, we manually characterized sleep and wake behavior for the first four hours of a single active and inactive period (see Methods). As a positive control to validate our analysis system, we reasoned that animals would show relatively more sleep during the inactive period. Our results (Fig. 2a) clearly show a strong effect of period with both young and aged animals spending significantly more time sleeping in the inactive period, and no significant difference in total sleep time with age.

Prior studies have reported a loss of the deep slow wave sleep component with age in both rodents and humans [12], [41], [42], [43], [44] although see reports [45], [46]. To evaluate age-related changes in deep sleep, we segregated the first four hours of the inactive period, a time frame reportedly enriched in deep slow wave sleep [47], [48], into component wake, light, REM, or deep (slow wave) sleep (see Methods). The percentage of total time spent in each identified stage is plotted (Fig. 2B) and shows a significant age-related increase in light sleep at the expense of deep sleep.

**Figure 3:**
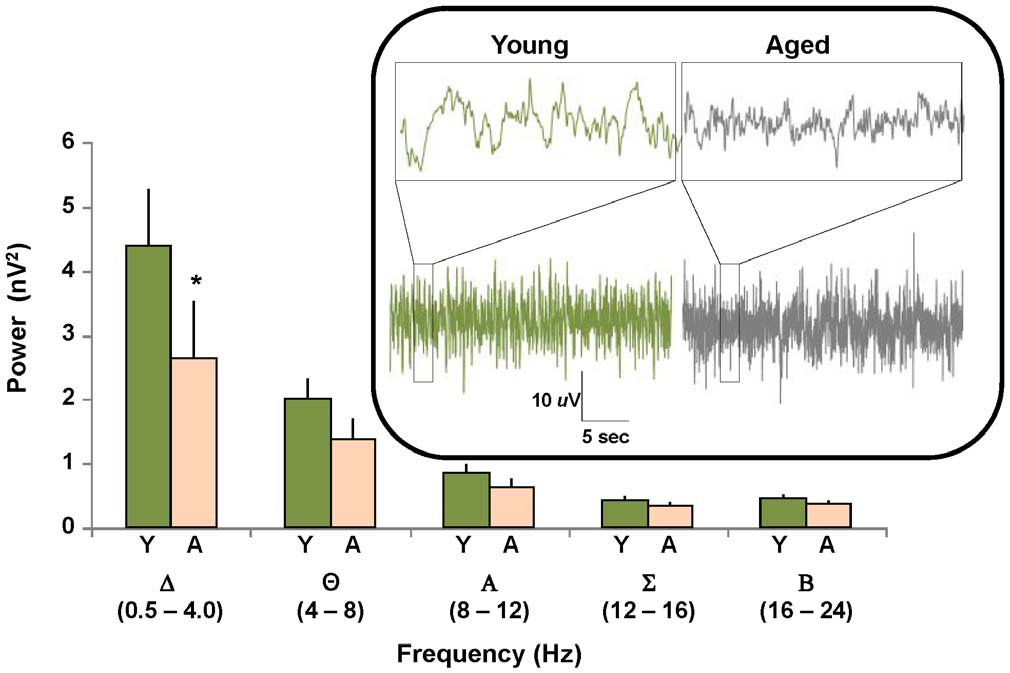
Loss of deep sleep delta power with age. Averaged power of delta, theta, alpha, sigma, and beta frequencies (x axis) are plotted as a function of age. Delta, the dominant hallmark frequency of deep sleep, is significantly reduced with age (2- ANOVA repeated measures; p<0.001 main effect of frequency; p = 0.08 main effect of age; p = 0.03 interaction term; * p<0.05 post-hoc Tukeys test). **Inset:** Representative EEG traces from young and aged subjects during deep sleep (upper detail- 1 s window) depicts reduced large amplitude, slow wave activity with age.

We next performed a power analysis investigating the intensity of deep sleep during the inactive period. The first four bouts of inactive period deep sleep (‘bout’ defined as ≥2 min. uninterrupted deep sleep) from each subject were analyzed. Fast Fourier EEG transforms were used to calculate power for each of 5 frequency ranges: Δ (0.5–4 Hz), Θ (4–8 Hz), Α (8–12 hz), Σ (12–16 Hz) and B (16–24 Hz). Results were averaged within each frequency range across all four bouts for each animal and treated as a single observation. Results are plotted and analyzed as a function of age (Fig. 3- spectral analysis). Deep sleep's large Δ component was significantly and selectively decreased in aged subjects. Taken together, these results show that inactive period deep sleep duration and intensity (power) were reduced with age.

#### Relationship between deep sleep and water maze performance

**Figure 4:**
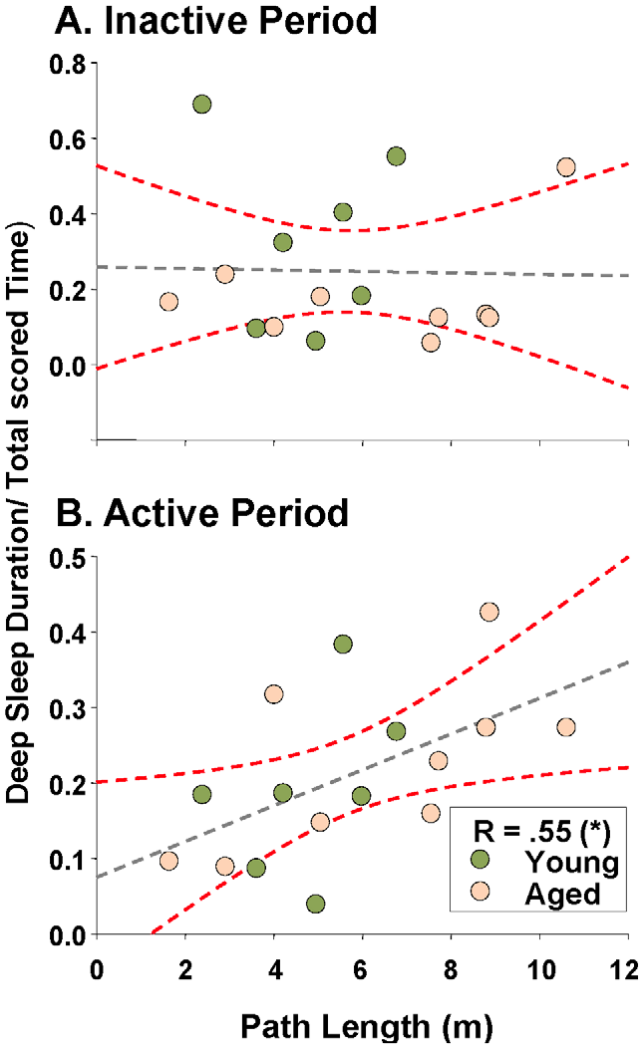
Maze performance is not correlated with duration of deep sleep during the inactive period (A. p>0.9), but worsens with increased active period deep sleep (B. p = 0.023; Pearson's test). The proportion of deep sleep duration divided by total time scored (y axis) is plotted as a function of average training path length (x axis). Animals that experienced more deep sleep during the active period tended to have increased path lengths (worsened performance).

Because a significant inactive period deep sleep loss paralleled age-related behavioral deficits in the Morris water maze, we hypothesized that as deep sleep was lost, maze performance would worsen in individual subjects. Both deep sleep duration and power were tested against water maze latency and path length. Contrary to our prediction, all results were non-significant (e.g., Fig. 4A p>0.9, Pearson's correlation between inactive period deep sleep and training trial path length). Interestingly, there was a significant correlation between increasing maze training path length (worsening performance) and increased *active* period deep sleep (Fig. 4B- as active period deep sleep increased, maze performance worsened), suggesting that increased deep sleep during a normally wake-enriched period could be disruptive for maze performance. Further, if subdivided by age, the young subjects show no significant correlation between maze performance and active period deep sleep (R = 0.4; p = 0.29), while the aged subjects show a significant correlation (R = 0.63; p = 0.03), indicating that the overall correlation is primarily driven by the relationship between deep sleep and maze performance within aged subjects.

### Microarray Transcriptional Profile of Parietal Cortex

**Figure 5:**
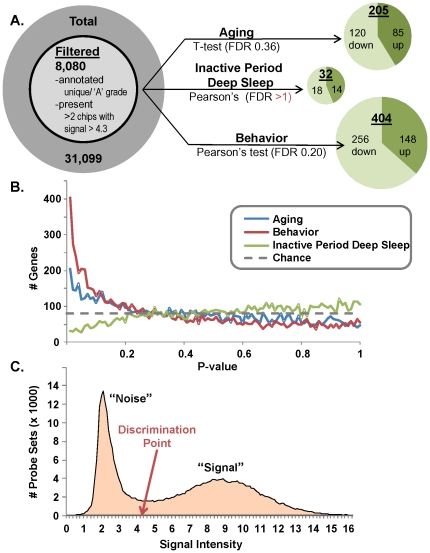
Microarray analysis overview. **A**. *Left:* Microarray filtering strategy. Probe sets with low quality annotations were removed. To address redundancy, if two ‘A’ grade probe sets claimed to represent the same gene symbol, only the probe set with the highest average signal intensity was retained. Among these, probe sets for which more than 2 arrays reported a signal intensity >4.3 were retained for analysis. *Center:* 3 tests were performed on the data- Aging, Behavior (correlation with Probe Trial latency), and Sleep (duration of deep sleep during the inactive period). Type of test used (α = 0.01 for all tests) and False Discovery Rate (FDR) are shown. *Right:* Significant results separated into up and downregulated for each test. Size of pie charts are roughly proportional to number genes found (note: sleep analysis finds fewer genes than expected by chance). **B**. P-value histograms for statistical results plots the number of genes found significant as a function of the p-value cutoff bin (0.01 increments) in which they were discovered. Both behavior (red) and aging (blue) show more genes than expected by chance (dashed gray line) at small p-values, while inactive period deep sleep (green) does not. **C**. Signal intensity frequency histogram depicts rationale for choosing 4.3 as a cutoff for presence calls. The number of probe sets (y axis) are plotted as a function of signal intensity (x axis). A narrow, low intensity ‘noise’ peak centered around 2.4, a broad high intensity ‘signal’ peak around 8.8, and a saddle region between the two from ∼3.5–∼5.5 are apparent. We selected the midpoint of that saddle region to discriminate signal-rich from noisy probe sets.

Because EEG electrode placement was over parietal cortex, gene transcriptional profiles were taken from the same region to more accurately align results from these two different measurement systems. Gene transcription of the parietal cortex was analyzed for aging, behavior, and deep sleep associated transcriptional influences (Fig. 5A). Parietal cortex was removed from a subset of subjects (n = 6/age group) and extracted RNA was hybridized to individual microarrays (Affymetrix RAE 230 v. 2). Probe sets that were not “A” grade annotated, unique, and present (i.e., had sufficient signal strength- Fig. 5C) were excluded from analysis. The remaining 8,080 genes were analyzed for effects of aging (pairwise t-test comparison between young and aged subjects), behavior (correlation with water maze probe latency- Fig. 1) and deep sleep (correlation with duration during the inactive period- Fig. 2B right). Water maze performance, and to a lesser degree aging, appear to have strong, statistically reliable transcriptional signatures. Conversely, deep sleep during the inactive period correlates with fewer genes than would be expected by chance (false discovery rate >1 for sleep in Fig. 5B). Because we observed a statistically significant correlation between maze performance and active period deep sleep, we also examined gene signatures associated with active period deep sleep (see ‘Genes associated with active period deep sleep gain’).

#### Aging Transcriptional Profile

205 genes changed significantly with age (α =  0.01; Fig. 5A). Of these, 85 were upregulated and 120 downregulated (see Table S1 for an alphabetical list of genes significant in at least one of the three analyses). Pathway level investigations of the aging signature identified lysosomal and immune upregulation, as well as synaptic pathway downregulation groups (Table 1).

**Table 1:** Selected functional processes identified by the DAVID overrepresentation functional clustering algorithm (see Methods) are shown for aging, behavior, and sleep related genes.

Ontology	GO ID	Description	#	p-value
**Upregulated with Age**				
CC	GO:0005764	lysosome	13	1.88^-09^
BP	GO:0019882	antigen processing and presentation	7	3.11^-07^
CC	GO:0022627	cytosolic small ribosomal subunit	4	0.00776
CC	GO:0005770	late endosome	4	0.00899
BP	GO:0030595	leukocyte chemotaxis	3	0.01653
**Downregulated with Age**				
BP	GO:0006950	response to stress	21	0.00206
MF	GO:0017076	purine nucleotide binding	25	0.00212
BP	GO:0032412	regulation of ion transmembrane transporter activity	3	0.01071
BP	GO:0052548	regulation of endopeptidase activity	4	0.02458
BP	GO:0048812	neuron projection morphogenesis	6	0.04123
**Increased as Animals Take Longer to Complete the Maze**				
BP	GO:0012501	programmed cell death	18	0.00200
CC	GO:0005773	vacuole	12	0.00899
BP	GO:0015031	protein transport	23	0.01110
BP	GO:0007049	cell cycle	18	0.01216
MF	GO:0003743	translation initiation factor activity	6	0.01289
BP	GO:0016052	carbohydrate catabolic process	6	0.02935
BP	GO:0006412	translation	13	0.03957
**Decreased as Animals Take Longer to Complete the Maze**				
BP	GO:0006816	calcium ion transport	6	0.00493
BP	GO:0050804	regulation of synaptic transmission	7	0.01093
CC	GO:0014069	postsynaptic density	5	0.02272
**Increased with Active Period Deep Sleep in Aged Animals**				
BP	GO:0006414	translational elongation	11	0.00010
BP	GO:0006412	translation	15	0.00373
CC	GO:0005840	ribosome	12	0.00344
**Decreased with Active Period Deep Sleep in Aged Animals**				
BP	GO:0042552	myelination	9	0.00002
MF	GO:0046943	carboxylic acid transporter activity	8	0.00080
BP	GO:0006643	membrane lipid metabolic process	6	0.00806
CC	GO:0005856	cytoskeleton	30	0.01336
BP	GO:0008088	axon cargo transport	4	0.01388
CC	GO:0043005	neuron projection	19	0.04293

The filtered gene list (8080 genes) was used as background, and each of the six gene lists (Up or Downregulated with age; Increased or Decreased as a function of latency in the water maze; Increased or Decreased as a function of active period deep sleep duration) were analyzed separately in the Gene Ontology. A single process (p≤0.05) from each cluster was selected. *Abbreviations*: GO ID- Gene Ontology Accession ID; CC- cellular component; BP- biological process; MF- molecular function; #- number of genes significant in category; p-value- DAVID statistical test result.

Upregulated aging-identified processes appeared similar to those found in other rodent brain aging microarray studies, particularly antigen presentation/inflammatory and lysosome/endosome related pathways [27], [28], [29], [49], [50], [51], [52], [53], [54], [55]. Downregulated processes reflected a blunted stress response and cell signaling (purine nucleotide binding, ion transport, regulation of endopeptidase activity- possibly related to the stress response). However, there was a notable lack of downregulated aging processes related to neurons (with the exception of neuron projection morphogenesis) as compared to prior hippocampal aging studies [27], [28], [29], [30], [52], [53].

**Figure 6:**
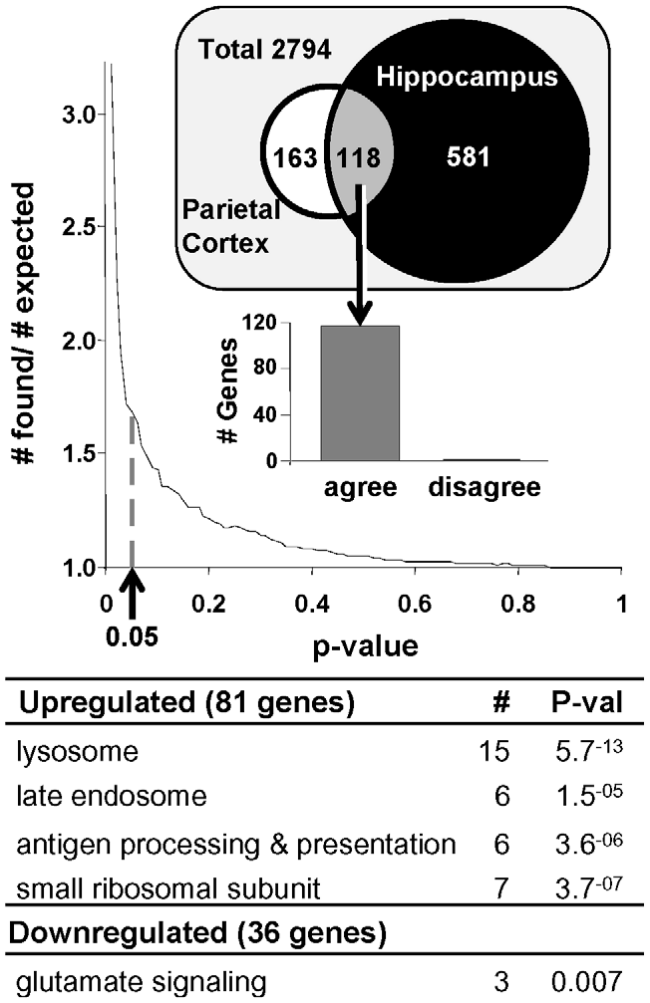
Similarity in aging transcriptional profiles across brain regions. The aging transcriptional signature for parietal cortex was contrasted with a prior study examining the transcriptional profile of aging in the hippocampus (Kadish et al., 2009). The proportion of genes found in the overlap divided by the number expected by chance in the overlap (y-axis) is plotted as a function of p-value cutoff (x-axis). We selected a p = 0.05 cutoff (arrow- dashed line) for our overlap analysis. **Venn diagram:** Out of 2794 total genes present in both studies, far more were significantly changed with age in hippocampus. Of the genes changed with age in parietal cortex, nearly half were also identified in hippocampus. **Right:** 117/118 overlapping genes agreed in direction (up or downregulated in both studies). **Lower:** DAVID analysis of common age-regulated genes. Functional group name, number of genes (#) and probability that number of genes in that category would be identified by chance (p-value) are shown.

To formally test for similarity, we directly compared the aging transcriptional profiles of parietal cortex (present study) and hippocampus [28] at the gene, rather than the pathway, level. Only genes that were present and annotated in both studies (2794 genes) were evaluated. To determine a single Type I error cutoff (α level) for both studies, we constructed fold-enrichment graph (Fig. 6, upper) depicting the relative increase over chance discovery that real data comparisons show [as in [32]]. At a p-value cutoff of 0.05 (Fig. 6, dashed line), there appears to be a sharp upturn in fold-enrichment, indicating that genes assigned a p-value≤0.05 in both studies begin to show strong agreement with one another. Thus, the Type I error was set at α = 0.05 for comparison across the two studies.

Parietal cortex yielded 281 significant genes and hippocampus 699 (Fig. 6, Venn diagram). 118 genes were common between the two studies. The probability of finding a certain number of genes in the overlap by chance can be estimated based on % significant genes in parietal cortex aging and % significant genes in hippocampal aging. The product of these two percentages gives the percentage of genes that should be found in the overlap (parietal cortex: 10% (281/2794) * hippocampus: 25% (699/2794)  = OVERLAP: 2.5% (or 70 genes). Here, we expect 70 genes and find 118- the actual number of genes in the overlap exceeds that expected by chance and was highly significant (p = 3.6^-8^, binomial test). Further, the majority of overlapping genes (117/118) agree in direction (Fig. 6, upper- inset) and, as reviewed above, reflect increased lysosomal and inflammatory processes with aging (Fig. 6, lower).

#### Behavioral transcriptional profile

404 genes were significantly correlated to latency on probe trial (Pearson's test, p≤0.01). 148 significant genes with negative R values showed reduced expression, and 256 with positive R values showed increased expression, as maze performance worsened (Fig. 5A). Functional grouping analysis (Table 1) shows increased transcriptional activity related to apoptosis (programmed cell death, cell cycle), macromolecular synthesis (translation initiation factor, protein transport, translation), and energy (carbohydrate catabolism). Downregulated categories appeared to strongly represent the neuronal compartment. Although genes in these categories were negatively correlated with latency on the water maze (their mRNA levels decreased as performance on the maze worsened), they were not significantly altered with age. It is interesting to note that the majority of behavior-identified genes (395/404; 98%) showed the same direction of change with age (negatively correlated genes were decreased with age, positively correlated behavior genes were increased). Although most were non-significant with age, this ‘directional agreement’ appears highly unlikely to be a chance occurrence. Assigning a simple binomial test 50% probability of up or downregulation (that is- by chance assuming that any gene selected by behavioral correlation has a 50% chance of being up- or downregulated by age) yields p<1^−12^ likelihood such directional agreement could have occurred by chance.

#### Relationship between aging and behavioral profiles

Based on the observations above, we performed an overlap analysis of aging and behavior significant genes. Twenty one genes were significant (p≤0.01) in both tests, and all agreed in direction (e.g., genes positively correlated with latency on the Morris water maze were increased with aging). By chance, 10 genes would be expected in the overlap. This small but significant set of genes include:

**Upregulated:** *Trem2* (Triggering receptor expressed on myeloid cells 2; may participate in activation of the immune response), *RT1-Aw2* (Class I histocompatibility antigen, Non-RT1.A alpha-1 chain; presentation of antigens to immune cells), *H2-M3 (*MHC class I antigen H-2M3), *Sult1a1* (Sulfotransferase 1A1), and *Cngb1* (Cyclic nucleotide-gated cation channel beta-1).
**Downregulated:** *Ccr5* (chemokine (C-C motif) receptor 5; receptor for inflammatory cytokines), *Dync2h1* (dynein cytoplasmic 2 heavy chain 1; involved in intracellular transport), *Dmgdh* (dimethylglycine dehydrogenase), *Neto2* (neuropilin [NRP] and tolloid [TLL]-like 2; receptor accessory subunit that increases kainate receptor activity), *Necab3* (N-terminal EF-hand calcium binding protein 3; a promoter of beta amyloid formation), *rCG_32844* (ubiquitin specific protease 43), *Emid1*(EMI domain containing 1), *Acss2* (acyl-CoA synthetase short-chain family member 2; activates acetate for lipid synthesis or energy production), *Serp1* (stress-associated endoplasmic reticulum protein 1; protects unfolded proteins in the ER from degradation), *Igf1* (insulin-like growth factor 1; similar to insulin with greater growth promoting activity), *Alcam* (activated leukocyte cell adhesion molecule; cell adhesion molecule involved in neurite extension), *Foxk2* (forkhead box K2; transcription factor that binds NFAT-like motifs in the interleukin 2 receptor coding region and can inhibit NFAT-mediated upregulation of cytokines), *Pdpk1* (3-phosphoinositide dependent protein kinase-1; phosphorylates and activates PKB/AKT, PKA, PKC-zeta, RPS6KA1 and RPS6KB1), *Nupl1* (nucleoporin like 1; Component of the nuclear pore complex, a complex required for the trafficking across the nuclear membrane), and *Ctsk* (cathepsin K; may play a role in extracellular matrix degradation).

By this analysis, 2.1x as many genes as would be expected by chance (21/10) were found in the overlap between behavior and aging. The same analysis in the Kadish data set (isolated young and aged subjects only) revealed 231 genes common to both aging and behavior, while only 31 would be expected by chance (6.75x increase over chance). The relationship between aging and behavioral transcriptional profiles appears much stronger in hippocampus than parietal cortex. This is consistent with hippocampus' reportedly more direct role in water maze performance and enhanced vulnerability to aging [56], [57].

#### Genes associated with inactive period deep sleep loss

Although we clearly saw a loss in both the power and the duration of inactive period deep sleep (Figs. 2 and 3), correlation analyses with transcriptional profiles did not identify a statistically reliable transcriptional signature in parietal cortex (Fig. 5a and b- nor was there a reliable correlation signature among aged animals alone- data not shown). Data mining analyses against a prior ‘extreme groups’ rodent hippocampal aging microarray study (impaired vs. unimpaired cognitive aging n = 20 F344 per group; hippocampal microarray- similar to the overlap analysis in Fig. 6) [27], did not reveal any significant similarity between sleep- and cognition- related genes in the two studies. However, significant genes from this analysis are included in Table 1 as this small subset could represent plausible candidates if replicated in future studies.

#### Genes associated with active period deep sleep gain

Because there was a significant, positive correlation between increased active period deep sleep and worsening maze performance (increased training path length), we also looked at transcriptional signatures correlated with this sleep behavior. The number of genes correlated with active period deep sleep did not exceed chance (∼404 genes expected at α = 0.05, 431 genes found; FDR = .94- data not shown). If partitioned by age, young subjects showed a profile with an FDR >1, while aged animals showed a statistically more reliable profile (∼404 genes expected, 560 genes found, FDR = 0.72; Table S2). Increasing the α stringency did not improve this relationship although relaxing it did worsen the FDR. As has been noted in prior work [58], pathway-level information can be more reliable than gene-level information, at least in part because of a ‘winnowing’ effect (false positives are less likely than true positives to participate in similar pathways). Intriguingly, the functional overrepresentation analysis of this set of genes did identify upregulated translational and downregulated neuronal/myelin pathways (Table 1, lower) that are consistent with current hypotheses regarding slow wave function in deep sleep.

Standardized tools (such as the DAVID suite of bioinformatic utilities used to help create Table 1 [59]) greatly facilitate identification of overrepresented functional pathways. However, assigning a probability to the likelihood that identified pathways support *a priori* hypotheses (like those related to slow wave sleep) requires a different approach. We re-examined DAVID output (note that the Functional Annotation Clustering option is used throughout DAVID analysis to reduce gene-level redundancy, an important consideration here). For positive correlations, there were 69 total gene pathways (clusters) identified by DAVID, 3 of which were significant. We then marked all 69 clusters as either supporting (6 clusters) or not supporting (63 clusters) macromolecular synthesis based on annotation content. 3/69 were significant (∼4%), 6/69 were hypothesis-related (∼9%), and the probability that a single pathway would be both significant and hypothesis-related is (9% * 4% = 0.4%). Overall, finding 3 significant and hypothesis-related pathways is highly unlikely (p = 0.0002; binomial test). Results for a similar analysis of downregulated pathways were: 89 total pathways (clusters): 6 (∼7%) significant; 10 hypothesis-related (associated with neuronal or myelin-related function, ∼11%); probability that 5 significant and hypothesis supporting pathways would be found by chance was highly unlikely (p = 7^-5^, binomial test).

## Discussion

### Sleep and cognition

A deficit in water maze performance reported here confirms numerous prior studies supporting the rat as a model of human age-related cognitive decline (reviewed in [60]). The observed loss of inactive period deep sleep duration and power with age is also commonly seen in humans [12], [41]. However, in rodent aging, researchers have variably reported no change in sleep [61], reduced or fragmented sleep [46], or changes in REM sleep [45], [62], [63]. Our results lend support to prior rodent studies [42], [43], [44], [64] showing deep, slow wave sleep loss during the inactive period with age. Discrepancies regarding sleep measures in prior studies may be related to different stress levels, a well understood disruptor of sleep [65]. Further, issues such as electrode placement and analysis technique could influence results. For instance, our results show that deep sleep's loss is light sleep's gain during the inactive period. This “see-saw” effect may have been missed in studies combining light and deep sleep into a single ‘non-REM’ category [42], [43]. Finally, the significant decrease in inactive period deep sleep was correlated with neither behavioral nor transcriptional profiles to an appreciable degree, contrary to our initial hypotheses.

Behavioral deficits did correlate significantly with *active* period deep sleep increases, particularly within the aged subjects. Prior work has shown that failed synaptic potentiation, and/or enhanced depotentiation corresponds to poor memory acquisition and is worsened with age [66], [67], [68], [69], [70]. Because two current hypotheses regarding the function of slow wave sleep (synaptic depotentiation [23], [24]; macromolecular synthesis [25]) could both conceivably contribute to this poor memory acquisition, we speculate that these deep sleep, slow wave driven processes, if engaged during the active rather than inactive period, may be disruptive to ordinary learning and memory processes. Regardless, understanding the mechanistic link between active period slow wave sleep and cognitive decline in aged rats may have important implications for understanding the role and consequences of breakthrough sleep, napping, and excessive daytime sleepiness on cognitive deficits in aging humans [41]. Although generally not as well studied, it is interesting to note that there are parallels in the human population, where complaints of nighttime (inactive period) sleep disturbances are more common, but excessive daytime sleepiness more strongly predicts poor mental function [71] and has been associated with increased risk of age-related cognitive decline, dementia, and neurodegenerative disease [72], [73].

### Microarrays

Transcriptional profiles of parietal cortex were interrogated for age, behavior, and sleep related gene expression. The aging profile was similar to that found in prior array studies. At the pathway level, upregulated inflammatory and endosomal processes appear consistent in rodent aging across multiple labs, array platforms, and brain regions [27], [28], [29], [49], [50], [51], [52], [53], [54], [55], [74] (although see [75]). Processes downregulated with aging have been more variable across brain regions, possibly because of regional differences in age-related selective neuronal vulnerability [56]. Although we report downregulation of genes associated with stress response and cell signaling pathways in aging parietal cortex, there was a notable lack of downregulated processes related to neurons (with the exception of neuron projection morphogenesis) in contrast to consistent findings across prior hippocampal aging studies [27], [28], [29], [30], [52], [53]. Further studies examining neuron [76] and other brain cell-type specific array signatures [77] may help to address these concerns. Nonetheless, our analyses juxtaposing aging parietal cortex to published hippocampal array data with the same rat strain, gender, age range, and microarray chip design yielded strong and significant validation (Fig. 6).

Behavior alone explained more of the variability in gene expression than either aging or sleep measures. As performance in the water maze worsened, the expression levels of genes related to synaptic structure and Ca^2+^ homeostasis declined. These findings support prior work attributing age-related cognitive deficits to reduced synapse number (decreased post-synaptic densities) [57], [70], [78], [79] and Ca^2+^ dyshomeostasis, a key and well-supported hypothesis of neuronal dysfunction with aging [80], [81], [82], [83], [84], [85]. We also hypothesized that the relationship between behavior and age-related gene expression would be weaker in parietal cortex than hippocampus (a brain region known to be important for Morris water maze performance). We tested this by evaluating the number of genes whose expression levels were both significantly changed with aging as well as correlated with behavior. There was a 2-fold enrichment in parietal cortex, and a 6-fold enrichment in prior hippocampal studies. While both enrichments are significant, these data support the conclusion that aging gene signatures in the hippocampus appear more strongly related to water maze performance than those in the parietal cortex.

We tested the hypothesis that deep sleep and gene expression measures taken from parietal cortex would correlate. Parallel to behavioral associations, gene profiles were not related to inactive period deep sleep, but were tied to active period deep sleep, particularly among aged subjects. Unlike the behavior/aging signature, no prior work exists with which to validate this signature. Therefore, results are provided separately (Table S2) and should be interpreted with care. Intriguingly, despite these cautions, it is interesting to note that, among this set of active period deep sleep correlated genes, increased expression profiles related to translation and decreased profiles related neuron/myelin processes lend support to both the macromolecular synthesis [86] and synaptic depotentiating [23] hypotheses of deep sleep function. As has been noted in prior work [58], pathway-level information can be more reliable than gene-level information, at least in part because of a ‘winnowing’ effect (false positives are less likely than true positives to participate in similar pathways).

### Caveats

This study likely represents a small piece of a much larger puzzle on sleep and cognition with aging involving other brain regions (e.g., hippocampus, hypothalamus), cell-types (e.g., astrocytes, neurons), and sleep stages (e.g., REM, light sleep- including temporal isolation of tissue during sleep stages [25], ). Although there are several positive and new findings presented here, we also conclude that inactive period deep sleep, as measured from parietal cortex, does not correlate with maze performance or gene expression. Further studies increasing the number of subjects, or employing an alternative ‘extreme groups’ experimental design (for example see [27], [90], [91]- separating aged subjects into impaired and unimpaired based on behavior) may reveal this to be a false negative. However, our analyses comparing results to prior ‘extreme groups’ data (see Results- genes associated with inactive period deep sleep loss) did not demonstrate any significant trends. Additionally, the present study was sufficiently powered to detect age-related maze deficits, deep sleep loss, transcriptional alterations, and even an intriguing correlation with active period deep sleep. Taken together, these observations suggest the more likely scenario that age-related maze performance deficits are more directly tied to active period deep sleep gain than to inactive period deep sleep loss.

### Summary

The F344 rat models some common sleep architecture and cognitive changes seen with age in humans, including the cognitively disrupting influence of active period deep sleep. Microarray analysis suggests that the molecular processes, as far as they can be appreciated by mRNA measurement, engaged by active period deep sleep are consistent with the macromolecular and synaptic functions that have been ascribed to deep sleep. Thus, we propose that active period deep sleep is temporally misaligned but mechanistically intact in age. This leads us to the following observations/conjectures. First, it appears that aged brain tissue is capable of generating the slow waves necessary for deep sleep, albeit at a weaker intensity than in young. Second, this activity, presented during the active period, appears disruptive rather than beneficial to cognition. Third, it is possible that this active period deep sleep is a cognitively pathologic attempt to recover age-related loss of inactive period deep sleep. Finally, therapeutic strategies aimed at reducing active period deep sleep (e.g., by promoting active period wakefulness and/or inactive period deep sleep) may be highly relevant to cognitive function in the aging community.

## Supporting Information

Table S1 622 genes significant by at least one of three statistical tests (ttest- young vs. aged; Pearson's correlation - Morris water maze behavior; Pearson's correlation- Inactive period deep sleep duration) are listed in alphabetical order. Probe set ID (Affymetrix), Gene Title, Young and Aged average and SEM are also given, along with test p-values and R-values (where appropriate). Significant p-values (≤0.01) are color coded red (for age upregulated, or behavior positive, or inactive period deep sleep negative) or blue (for age downregulated, behavior negative, or inactive period deep sleep positive).
(XLS)

Table S2 560 genes significantly correlated (p≤0.05; Pearson's test) with active period deep sleep. These are listed in alphabetical order by gene symbol. Probe set ID (Affymetrix), Gene Title, R value, and p-value are also included.
(XLS)
